# A modified ovarian-adnexal reporting and data system ultrasound for differentiating ovarian carcinoma: integration of the risk of malignancy algorithm into the American College of Radiology’s O-RADS US framework

**DOI:** 10.3389/fonc.2026.1962862

**Published:** 2026-10-19

**Authors:** Mengze Liu, Dandan Li, Zhanqiang Jin

**Affiliations:** 1 Department of Ultrasound, Weifang People’s Hospital, Weifang, Shandong, China; 2 Department of Ultrasound, The Affiliated Nanhua Hospital, Hengyang Medical School, University of South China, Hengyang, Hunan, China

**Keywords:** CA125/HE4, ovarian carcinoma, ovarian-adnexal reporting and data system, risk of malignancy algorithm, ultrasound

## Abstract

**Introduction:**

Most ovarian cancer cases are diagnosed at advanced stages, primarily due to the limited efficacy of current classification systems that rely on imaging modalities with variable accuracy. Transvaginal ultrasound as an imaging modality is commonly achieved using the detection of morphological features, which has some limitations mainly related to sonographer experiences and subjective nature of the assessment. To address this limitation, we developed a novel scoring system by incorporating the risk of malignancy algorithm (ROMA) into the ovarian-adnexal reporting and data system ultrasound lexicon (O-RADS US) framework, aiming to refine ultrasound-based risk stratification.

**Methods:**

This retrospective study enrolled 151 patients (mean age, 51.6 years ± 15.3), with 159 ovarian-adnexal masses (median, 8.2 cm; range, 2.1-24.9cm) between January 2023 and May 2026. The masses were assessed according to O-RADS US criteria, while ROMA values were calculated preoperatively. Then, the modified O-RADS US system reclassified lesions by integrating ROMA-derived malignancy probabilities into O-RADS US categories.

**Results:**

The modified O-RADS US system demonstrated superior diagnostic efficiency for ovarian cancer detection, achieving an AUC of 0.922 (95% CI: 0.878–0.966), significantly outperforming both the original O-RADS US system (AUC = 0.815, 95% CI: 0.740–0.890; p < 0.05) and ROMA alone across premenopausal (AUC = 0.861, 95% CI: 0.812–0.910; p < 0.05), postmenopausal (AUC = 0.902, 95% CI: 0.860–0.943; p > 0.05), and combined cohorts (AUC = 0.894, 95% CI: 0.865–0.923; p > 0.05). A marked reduction in O-RADS 4 lesions and an increase in O-RADS 2/3 lesions, while O-RADS 5 lesions remained stable (23.3% vs. 23.9%), and the malignancy rate for Categories 4 and 5 lesions increased from 44.0% with the original system to 58.1% with the modified system.

**Conclusion:**

The incorporation of ROMA into the ACR O-RADS US framework significantly enhances diagnostic performance for ovarian carcinoma, and improves risk stratification accuracy, reducing unnecessary biopsies for low-to-moderate risk lesions.

## Introduction

Ovarian carcinoma (OC) comprises a heterogeneous group of malignancies characterized by diverse features such as etiology, molecular biology, and tissue of origin (1–4). It ranks as the fifth leading cause of cancer-related deaths among women in the Western world. Despite recent advancements in understanding OC biology—particularly epithelial subtypes—and improvements in treatment strategies like aggressive cytoreductive surgery and novel chemotherapeutic agents, mortality rates have remained largely unchanged over the past three decades. This stagnation is partly attributable to the absence of reliable early detection methods and the subtlety of initial symptoms, resulting in over 70% of cases being diagnosed at an advanced stage (Stage III/IV).

Transvaginal ultrasound (TVUS) is the most widely used imaging modality for evaluating adnexal masses, as it provides critical insights into malignant potential through the detection of morphological features not discernible on physical examination. However, the accuracy of TVUS assessments depends heavily on sonographer expertise and subjective interpretation. To address this limitation, standardized scoring systems have been developed to enhance diagnostic consistency. Among these, the American College of Radiology Ovarian-Adnexal Reporting and Data System ultrasound lexicon (O-RADS US) utilizes established sonographic criteria to categorize lesions (5). Nevertheless, the O-RADS US risk stratification system requires further clinical validation, particularly for Category 4 lesions, which exhibit a broad malignancy risk range (10%–50%). Additionally, the system’s reliability must be rigorously evaluated for both inter- and intra-observer variability, as its classification relies on subjective assessments of lesion characteristics.

Biomarkers represent a promising tool for differentiating benign from malignant ovarian tumors. The identification of reliable biomarkers for OC is critical for disease management and significantly impacts prognosis and patient survival. Carbohydrate antigen 125 (CA125) remains a widely used biomarker for OC; however, its clinical utility is limited by low specificity, as elevated levels may occur in non-malignant conditions such as menstruation, pregnancy, endometriosis, or peritoneal inflammatory disorders. Furthermore, factors including race, age, smoking, obesity, and prior hysterectomy may influence CA125 levels. To address these limitations, novel biomarkers such as Human Epididymis Protein 4 (HE4) have been investigated to enhance diagnostic specificity. Nonetheless, neither CA125, HE4, nor TVUS alone demonstrates sufficient sensitivity or specificity for early OC detection when applied singularly. Current evidence strongly supports the combined use of CA125 and HE4 as the most effective biological diagnostic strategy for OC (2, 6).

This rationale led to the development of the Risk of Ovarian Malignancy Algorithm (ROMA), which integrates CA125, HE4, and menopausal status to improve biomarker performance in stratifying ovarian cancer risk. Approved by the U.S. Food and Drug Administration for evaluating ovarian masses, ROMA has demonstrated utility as a preoperative diagnostic tool in multiple studies (7). However, conflicting evidence exists: a study by Jacob et al. reported no significant added benefit of ROMA over individual CA125 and HE4 measurements (8). Additionally, variability in diagnostic cut-off values and unclear superiority over existing methods have raised concerns about its clinical adoption (9–13). Consequently, this study seeks to evaluate an alternative scoring system against the widely utilized O-RADS US classification for ovarian mass assessment.

## Materials and methods

### Study patients

This retrospective study was approved by the Institutional Review Boards of Weifang People’s Hospital. Eligible participants included (1): patients diagnosed with primary ovarian-adnexal masses via transvaginal ultrasound (TVUS) who subsequently underwent surgical resection; (2) lesions classified according to the O-RADS US system; (3) available CA125 and HE4 data allowing for the calculation of the ROMA index; (4) diagnostic-quality ultrasound images; (5) no history of prior pelvic surgery; and (6) available post-surgical histopathological results. Participants were excluded based on the following criteria: (1) diagnosis of metastatic malignant ovarian-adnexal masses; (2) prior receipt of systemic therapy or surgery for the lesion; (3) typically benign lesions, including simple cysts or luteal hematoma; and (4) incompleteness of essential data or uncertainty regarding the final diagnosis. Between January 2023 and May 2026, a total of 151 consecutive patients with 159 ovarian-adnexal masses were enrolled in the final analysis. Study population diagram was shown in **Figure 1**.

**Figure 1:**
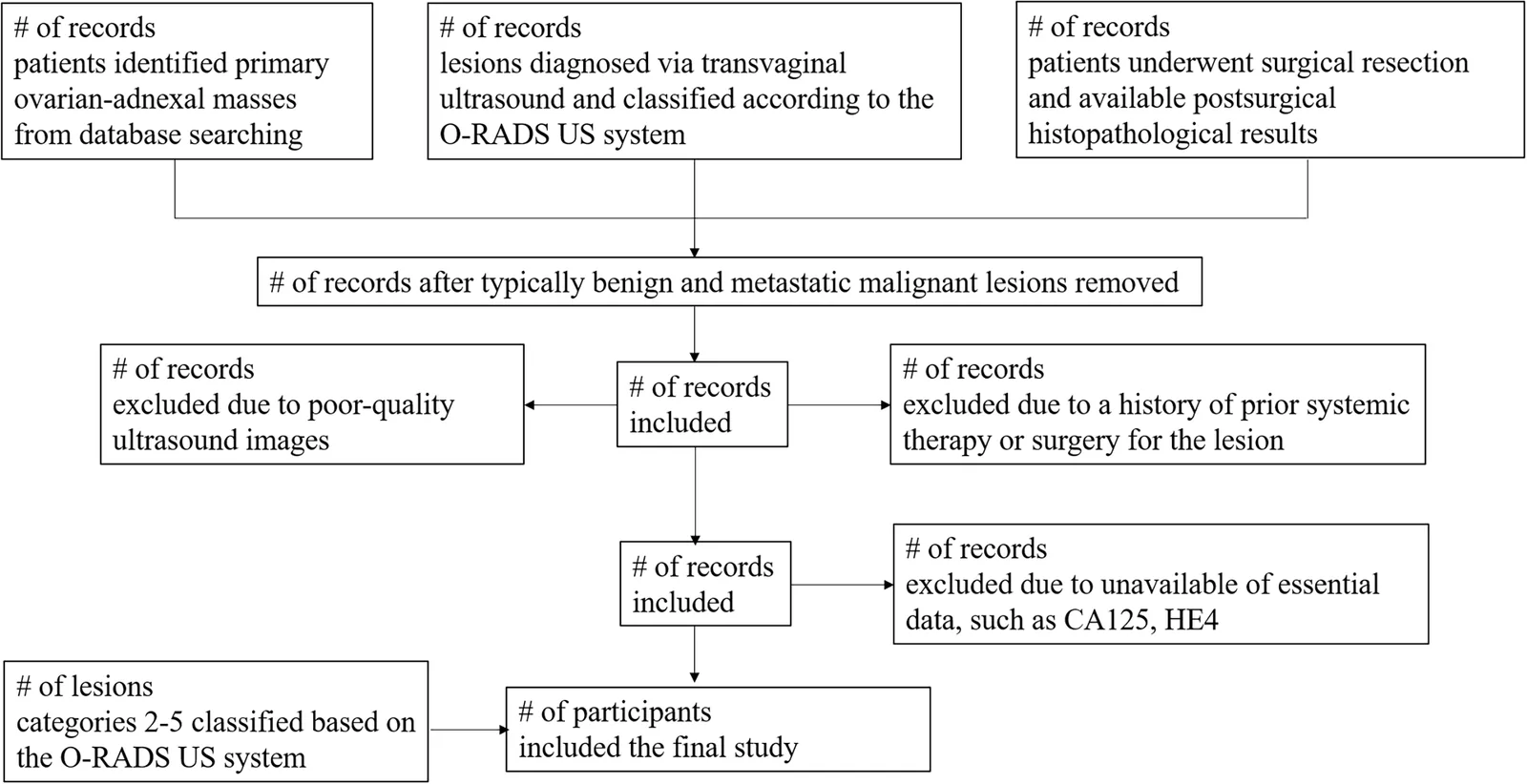
Study population diagram.

### Ultrasound protocols

TVUS scans of the ovarian-adnexal masses were obtained using a commercially available scanner (GE Voluson E 8/10 Medical Systems, Zipf, Austria) equipped with a 7- to 14-MHz linear phased-array transducer. For large lesions, assessment should be combined with transabdominal ultrasound. The following sonographic features were evaluated:

Component: cyst (unilocular or multilocular), solid, or complex;

Size: maximum diameter in three orthogonal planes;

External contour: smooth or irregular;

Internal contents: echogenicity (hypoechoic, isoechoic, or hyperechoic), calcifications, or acoustic shadowing;

Inner margin/walls: smooth, irregular (including complete/incomplete septations, solid components, or papillary projections), or calcifications;

Vascularity: absent, minimal, moderate, or marked vascular flow on Doppler imaging;

Ancillary findings: ascites, peritoneal thickening/nodules, or adenopathy.

Based on the O-RADS US guidelines, masses were classified into categories 2 to 5 to stratify malignancy risk. Two sonographers with more than a decade of experience in gynecological ultrasound independently evaluated the images and assigned O-RADS categories. Both were blinded to the initial O-RADS scores and the corresponding CA125, HE4, and ROMA values. Discrepancies between the two reviewers were resolved by a third senior examiner, and the final diagnosis was established once unanimous agreement was achieved. Both the ultrasound examination and biomarker tests were performed one week prior to surgery. Menopausal status was established based on the patient’s medical records.

### Tumor markers and ROMA

Blood samples were collected prior to surgery. Within four hours of collection, samples were centrifuged to isolate serum, which was then aliquoted into 5 mL cryotubes and stored at -20 °C. Serum levels of the biomarkers CA125 and HE4 were analyzed. CA125 concentrations were measured using the Architect CA125 II assay (Abbott Diagnostics, Abbott Park, IL), while HE4 levels were quantified via the HE4 EIA assay (Fujirebio Diagnostics, Malvern, PA). All biomarker analyses were conducted in the laboratory of Southern University of Science and Technology Hospital. Assays were performed strictly following the manufacturer’s protocols. The investigator responsible for biomarker analysis was blinded to clinical and pathological outcomes. ROMA value was calculated by using the Online ROMA Calculator on http://romatools.he4test.com/.

### Statistical analysis

Statistical analyses were conducted using SPSS 25.0 for Windows (IBM Corporation, Armonk, NY). Patient age is presented as mean ± standard deviation. Non-normally distributed data are reported as median and interquartile ranges. For bilateral lesions, a single value was selected per case for statistical analysis, ensuring that calculations were based on the number of patients rather than the number of lesions.

A receiver operating characteristic (ROC) curve was generated for the ROMA value to determine the optimal threshold for identifying ovarian-adnexal malignancy risk. The cutoff value (α) for ROMA was selected to maximize the combined sensitivity and specificity. ROC curves were also used to compare the diagnostic performance of two scoring systems:

Scoring system 1: Established O-RADS US categories based on TVUS features.

Scoring system 2: Combined O-RADS US categories and biomarker levels (TVUS features + ROMA), calculated as, scoring system 2 = O-RADS US + (ROMA – α)

Scoring criteria:

ROMA < α: Assigned -1

ROMA > α: Assigned +1

ROMA = α: Assigned 0

For each parameter, 95% confidence intervals (CIs) and the area under the curve (AUC) were calculated. A Z-test was performed to compare AUC values between scoring systems. All statistical tests were two-tailed, with significance defined as p < 0.05.

## Results

### Study population

The study cohort comprised 143 patients with unilateral ovarian-adnexal masses (143 lesions) and 8 patients with bilateral involvement (16 lesions). Among the 151 total patients, 103 (68.2%) were premenopausal and 48 (31.8%) were postmenopausal. The clinical characteristics of the 159 ovarian-adnexal masses are summarized in **Tables 1**, **2**.

**Table 1:** Histopathological findings of ovarian-adnexal masses.

Histopathological diagnosis	Number
Benign (n = 111)	
Ovarian serous cystadenoma	23
Ovarian mucinous cystadenoma	12
Ovarian fibroma	9
Ovarian endometriosis cyst	17
Ovarian follicular membrane cell tumor	5
Ovarian adenofibroma	3
Ovarian seromucinous cystadenoma	3
Struma-ovarii	2
Ovarian leiomyoma	9
Benign teratoma	23
Salpingitis/Sactosalpinx	5
Borderline (n = 11)	
Ovarian serous cystadenoma	8
Ovarian mucinous cystadenoma	2
Ovarian endometrioid tumor	1
Malignant (n = 37)	
High-grade ovarian serous carcinoma	11
Low-grade ovarian serous carcinoma	6
Ovarian granulosa cell tumor	2
High-grade ovarian mucinous carcinoma	2
Low-grade ovarian mucinous carcinoma	8
Ovarian clear cell carcinoma	2
Poorly/High differentiated endometrioid carcinoma	3/3

**Table 2:** Clinical characteristics of ovarian-adnexal masses.

Characteristics	Number	*p*
Nature (B/B/M, n)	111/11/37	–
Largest diameter (cm)		
Benign lesions	7.35 (4.9, 11.1)	
Borderline lesions	5.90 (8.5, 15.2)	
Malignancies	11.0 (7.3, 13.92)	0.008^*^
CA125		
Benign lesions	28.59 (13.15, 52.42)	
Borderline lesions	75.49 (40.72, 401.40)	
Malignancies	102.15 (52.5, 759.27)	0.000^*^
HE4		
Benign lesions	52.90 (42.54, 61.63)	
Borderline lesions	82.65 (47.10, 108.90)	
Malignancies	158.85 (91.94, 395.28)	0.000^*^
ROMA		
Benign lesions	14.70 (9.00, 25.50)	
Borderline lesions	37.40 (25.85, 63.77)	
Malignancies	91.20 (62.20, 139.50)	0.000^*^

^*^ Kruskal-Wallis test.

B/B/M, Benign/Borderline/Malignant.

### Lesion classification

Among the lesions:

Malignant: 37 lesions were identified in 33 patients (21.8% of the cohort, 33/151).

Benign: 111 lesions were identified in 107 patients (70.9% of the cohort, 107/151).

Borderline: 11 lesions were identified in 11 patients (7.3% of the cohort, 11/151).

The median CA125 and HE4 levels, along with the longest lesion diameters, differed significantly among malignant, borderline, and benign groups (p < 0.05).

### ROMA thresholds and diagnostic performance

ROC curve analysis identified ROMA thresholds for ovarian cancer (OC) detection as 29.70 in premenopausal patients and 42.50 in postmenopausal patients, with corresponding sensitivities and specificities of 80.6%/76.8% and 88.0%/78.5%, respectively.

The diagnostic performance of ROMA was higher in postmenopausal patients (AUC = 0.902, 95% CI: 86.0–94.3%) than in premenopausal patients (AUC = 0.861, 95% CI: 81.2–91.0%; z = 1.24 for between-group comparison).

For the combined cohort, the optimal ROMA threshold for OC identification was 42.80, achieving a sensitivity of 79.9%, specificity of 87.5%, and an AUC of 0.894 (95% CI: 86.5–92.3%) (**Figure 2**).

**Figure 2:**
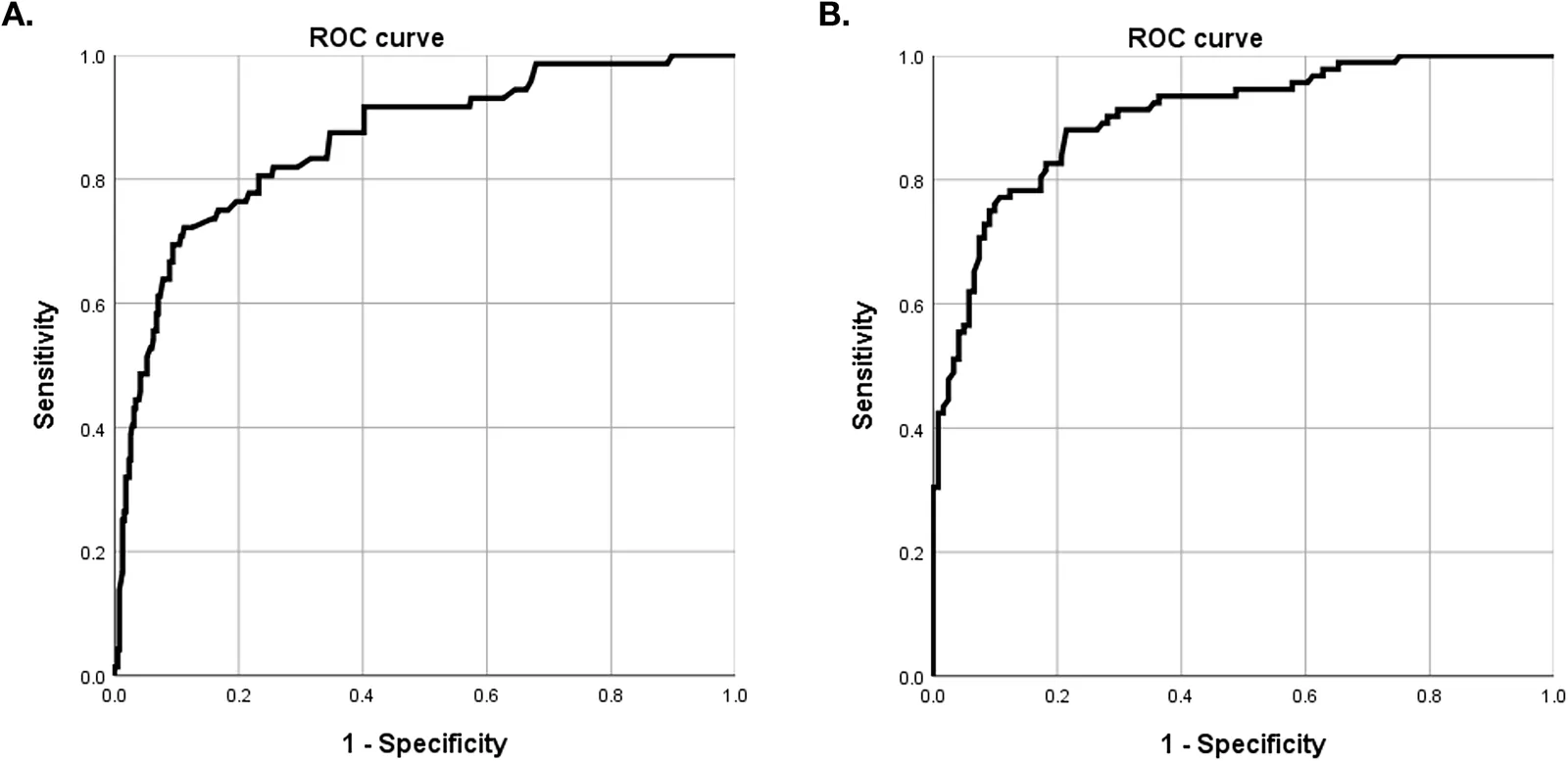
Receiver operating characteristic (ROC) curves for detection of malignancy of the risk of malignancy algorithm (ROMA) in premenopausal **(A)** and postmenopausal **(B)** patients, and the combined cohort **(C)**.

### O-RADS US vs. modified O-RADS US category distribution

The original O-RADS US classifications were distributed as follows: Category 2 (28.3%, 45/159), Category 3 (18.9%, 30/159), Category 4 (29.5%, 47/159), and Category 5 (23.3%, 37/159). In contrast, the modified O-RADS US classifications showed a distinct distribution: Category 2 (37.1%, 59/159), Category 3 (23.9%, 38/159), Category 4 (15.1%, 24/159), and Category 5 (23.9%, 38/159) (**Tables 3**, **4**).

**Table 3:** Comparison of the category of ovarian-adnexal masses between original and modified O-RADS US.

O-RADS US		Modified O-RADS US		Histopathology (n)		
Categories	n	Categories	n	Benign	Borderline	Malignant
2	45	OR 2	39	39	0	0
		OR 3	6	6	0	0
3	30	OR 2	20	20	0	0
		OR 3	6	6	0	0
		OR 4	4	2	2	0
4	47	OR 3	26	23	2	1
		OR 4	7	3	1	3
		OR 5	14	4	3	7
5	37	OR 4	13	4	3	6
		OR 5	24	4	0	20

**Table 4:** Detailed classification criteria of modified O-RADS US.

O-RADS US categories	Criteria	Modified O-RADS US categories
OR 2	If the lesion’s ROMA value exceeds the diagnostic threshold for ovarian cancer by 1% or more	OR 3
	It does not meet the aforementioned requirements	OR 2
OR 3/4	If the lesion’s ROMA value exceeds the diagnostic threshold for ovarian cancer by 10% or more	OR 4/5
	If the lesion’s ROMA value exceeds the diagnostic threshold for ovarian cancer by 1%-<10%	OR 3/4
	It does not meet the aforementioned requirements	OR 2/3
OR 5	If the lesion’s ROMA value exceeds the diagnostic threshold for ovarian cancer by 10% or more	OR 5
	It does not meet the aforementioned requirements	OR 4

According to the American college of radiology ovarian-adnexal reporting and data system ultrasound lexicon (O-RADS US), Category 2 lesions carry a malignancy risk of <1%, Category 3 lesions carry a risk of 1% to <10%, and Category 4 lesions carry a risk of 10% to <50%.

### Reclassification analysis

**Table 4** further details reclassification patterns (**Figures 3**–**6**).

**Figure 3:**
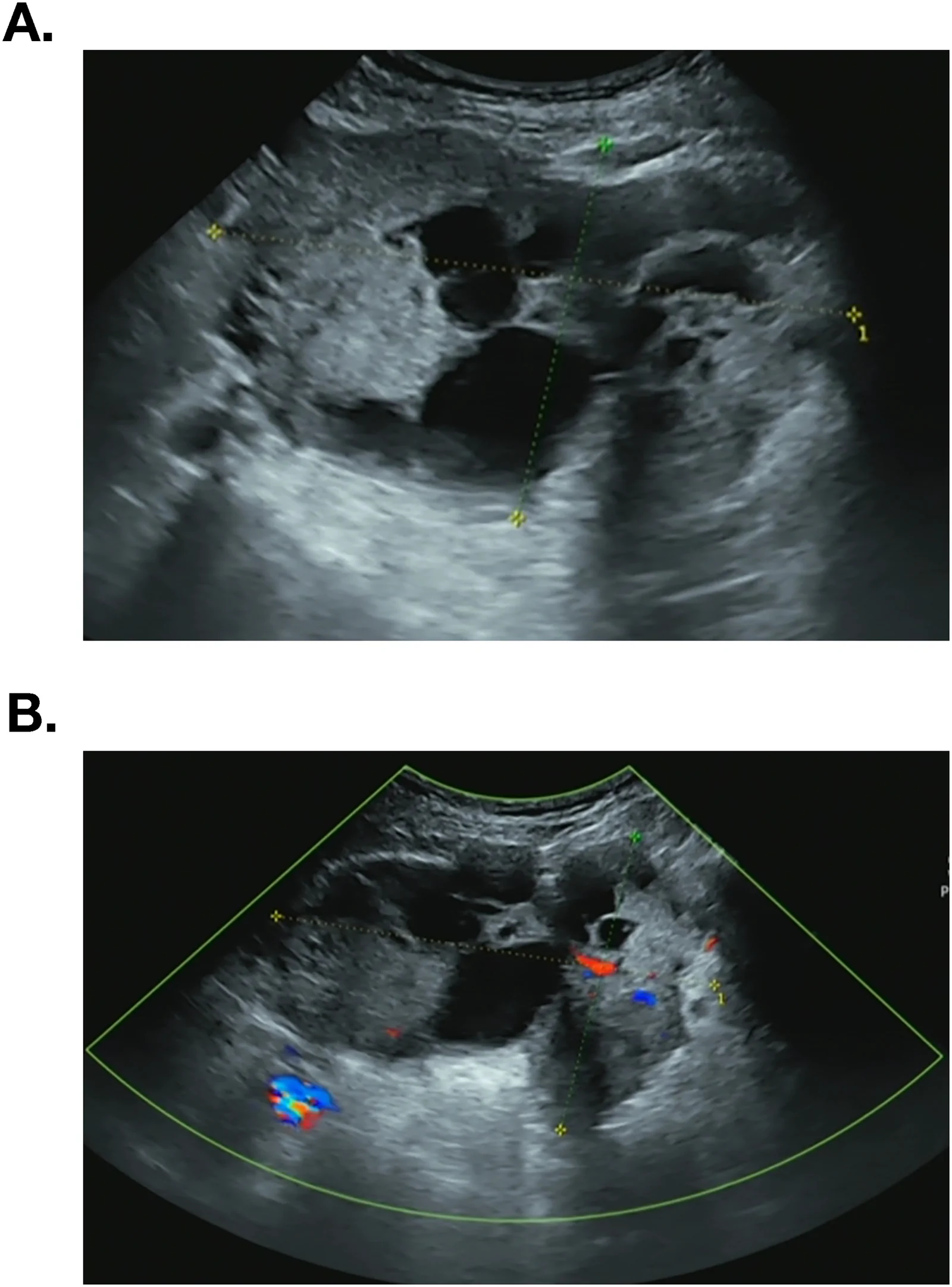
Multimodal evaluation of a complex ovarian mass in a 49-year-old premenopausal woman. Grayscale ultrasound: demonstrates a large multilocular cystic lesion measuring 12.5 cm in maximal diameter, containing internal solid components **(A)**. Color-doppler imaging: reveals minimal vascularity within the solid elements (color score: 2) **(B)**. Diagnostic workflow: initial O-RADS US classification: category 4 (based on multilocular cystic morphology with solid components and minimal vascularity). Biomarker profile: CA125: 208.9 U/mL; HE4: 279.1 pmol/L calculated ROMA: 84.3 (exceeding malignancy threshold of 29.7) Revised classification: upgraded to O-RADS US category 5 (driven by ROMA-based malignancy risk stratification). Histopathological diagnosis: postoperative analysis confirmed a high-grade serous cystadenocarcinoma, aligning with the biomarker-guided reclassification.

**Figure 4:**
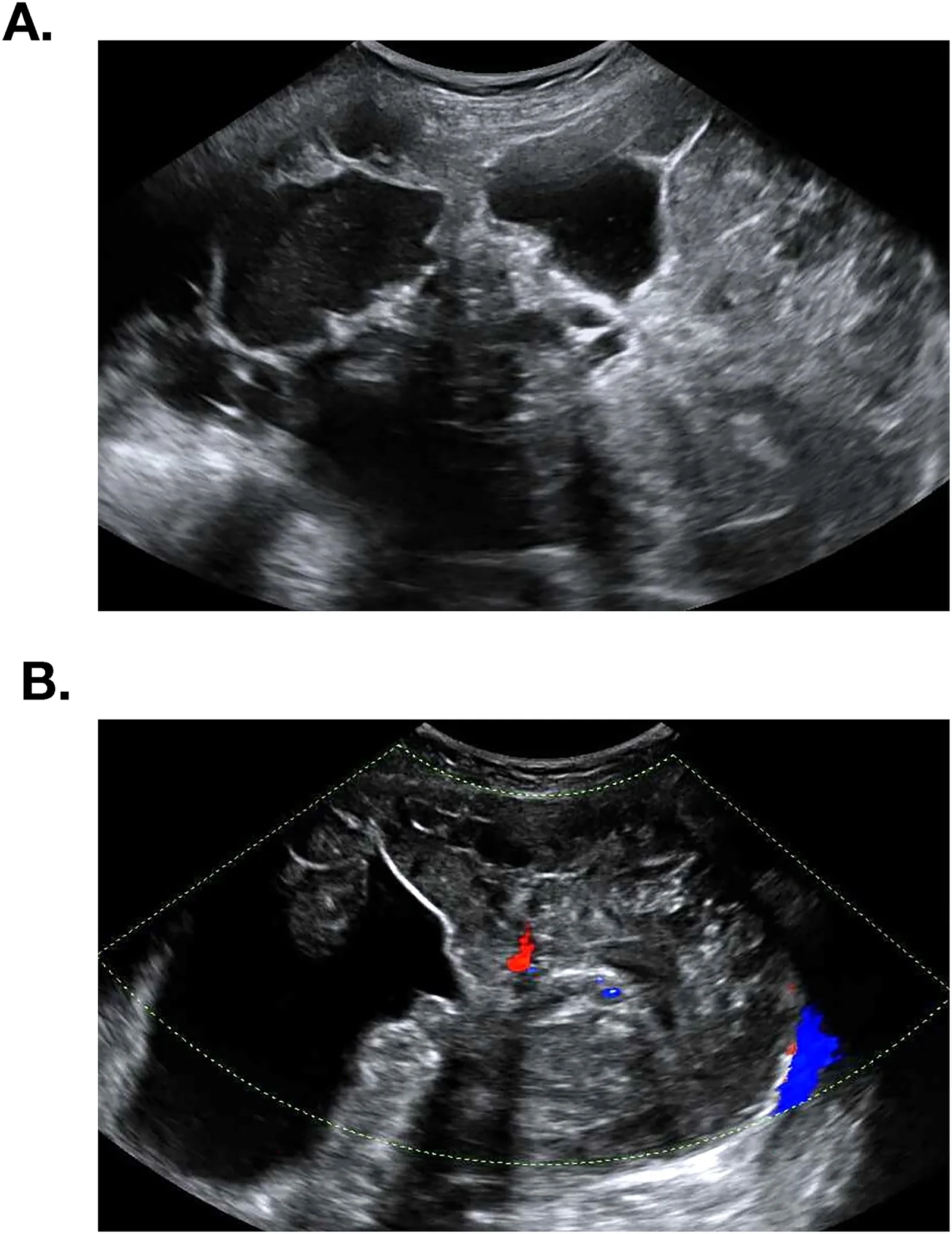
Multimodal assessment of an ovarian mass in a 68-year-old postmenopausal woman. Grayscale ultrasound: demonstrates a complex lesion measuring 39.0 cm in maximal diameter, containing internal cystic components **(A)**. Color-doppler imaging: reveals minimal vascularity throughout the lesion (color score: 1) **(B)**. Diagnostic workflow: initial O-RADS US classification: category 4 (based on complex morphology and vascularity). Biomarker profile: CA125: 1151.0 U/mL; HE4: 1005.0 pmol/L calculated ROMA: 98.6 (exceeding malignancy threshold of 42.5) revised classification: upgraded to O-RADS US category 5 (driven by biomarker-based risk stratification). Histopathological confirmation: final surgical pathology identified a borderline mucinous cystadenoma with partial transformation into mucinous carcinoma, consistent with the ROMA-guided classification upgrade.

**Figure 5:**
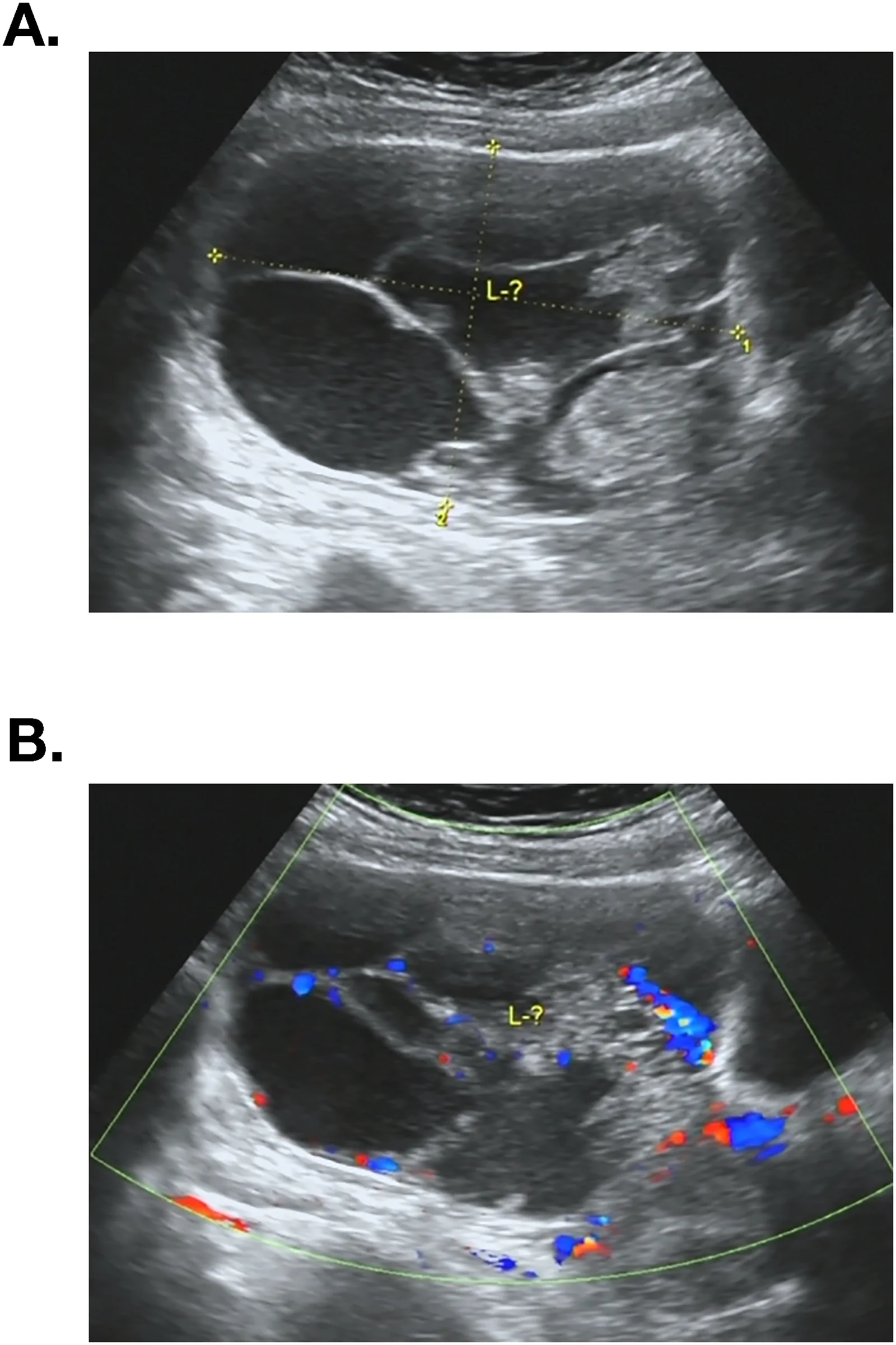
Ultrasound evaluation of a 39-year-old premenopausal woman with a complex ovarian mass. Grayscale ultrasound: demonstrates a multilocular cystic lesion measuring 10.5 cm in maximal diameter, containing solid internal components **(A)**. Color-doppler imaging: reveals minimal vascularity within the solid elements (color score: 2) **(B)**. Diagnostic workflow: initial O-RADS US classification: category 4 (based on moderate -risk features: large size, solid components, and vascularity). Biomarker profile: CA125: 52.4 U/mL; HE4: 165.0 pmol/L calculated ROMA: 59.9 (exceeding malignancy threshold of 29.7) revised classification: upgraded to O-RADS US category 5 (reflecting discordance between imaging and biomarker risk stratification). Histopathological diagnosis: final surgical pathology confirmed an endometrioid carcinoma, supporting the ROMA-guided reclassification.

**Figure 6:**
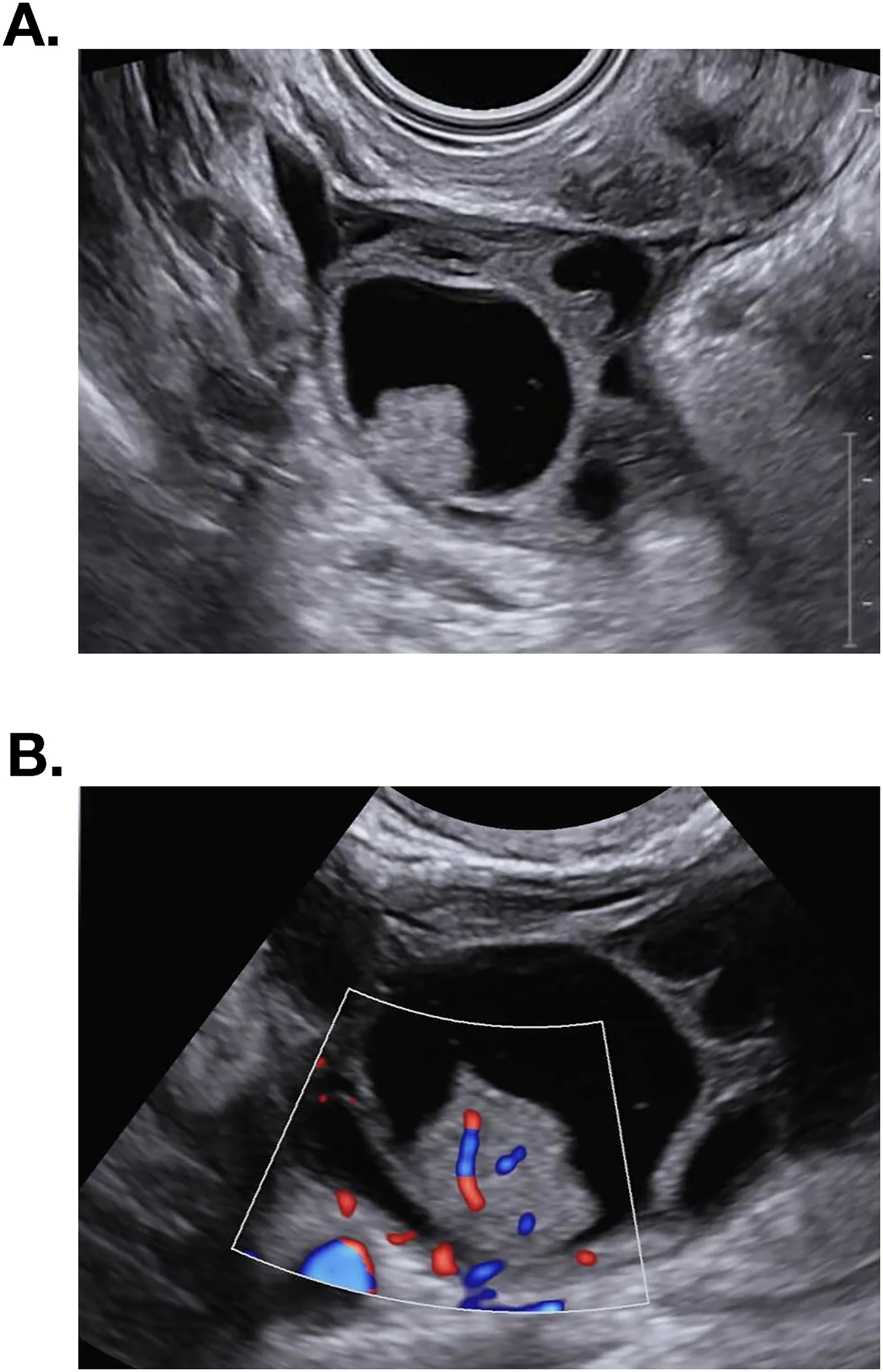
Ultrasound imaging findings in a 33-year-old premenopausal woman with an ovarian mass. Grayscale ultrasound demonstrates a unilocular cystic lesion measuring 2 cm in maximum diameter, characterized by papillary projections and irregular inner walls **(A)**. Color-doppler imaging reveals minimal vascular flow within the walls (color score: 2) **(B)**. Diagnostic workflow: initial O-RADS US classification: category 4 (based on sonographic features). Biomarker profile: CA125: 12.2 U/mL; HE4: 60.1 pmol/L calculated ROMA: 11.9 (below malignancy threshold of 29.7) revised classification: downgraded to O-RADS US category 3. histopathological correlation: postoperative analysis confirmed the lesion as a borderline serous cystadenoma, inconsistent with the ROMA-adjusted risk stratification.

Upgrades:

6 Category 2 lesions were reclassified as Category 3.

4 Category 3 lesions were reclassified as Category 4.

14 Category 4 lesions were reclassified as Category 5.

Downgrades:

13 Category 5 lesions were reclassified as Category 4.

26 Category 4 lesions were reclassified as Category 3.

20 Category 3 lesions were reclassified as Category 2.

### Diagnostic performance of scoring systems

Scoring system 2 demonstrated superior diagnostic efficiency for OC identification, with an AUC of 0.922 (95% CI: 0.878-0.966), compared to scoring system 1 (AUC: 0.815; 95% CI: 0.740-0.890; z = 2.43) and ROMA alone in both pre-/post-menopausal patients and the combined cohort. Using 3.5 as the optimal cutoff value, scoring system 1 predicted OC with 92.3% sensitivity and 60.0% specificity. In contrast, at a cutoff of 4.5, scoring system 2 achieved 82.1% sensitivity and 92.5% specificity (**Figure 7**).

**Figure 7:**
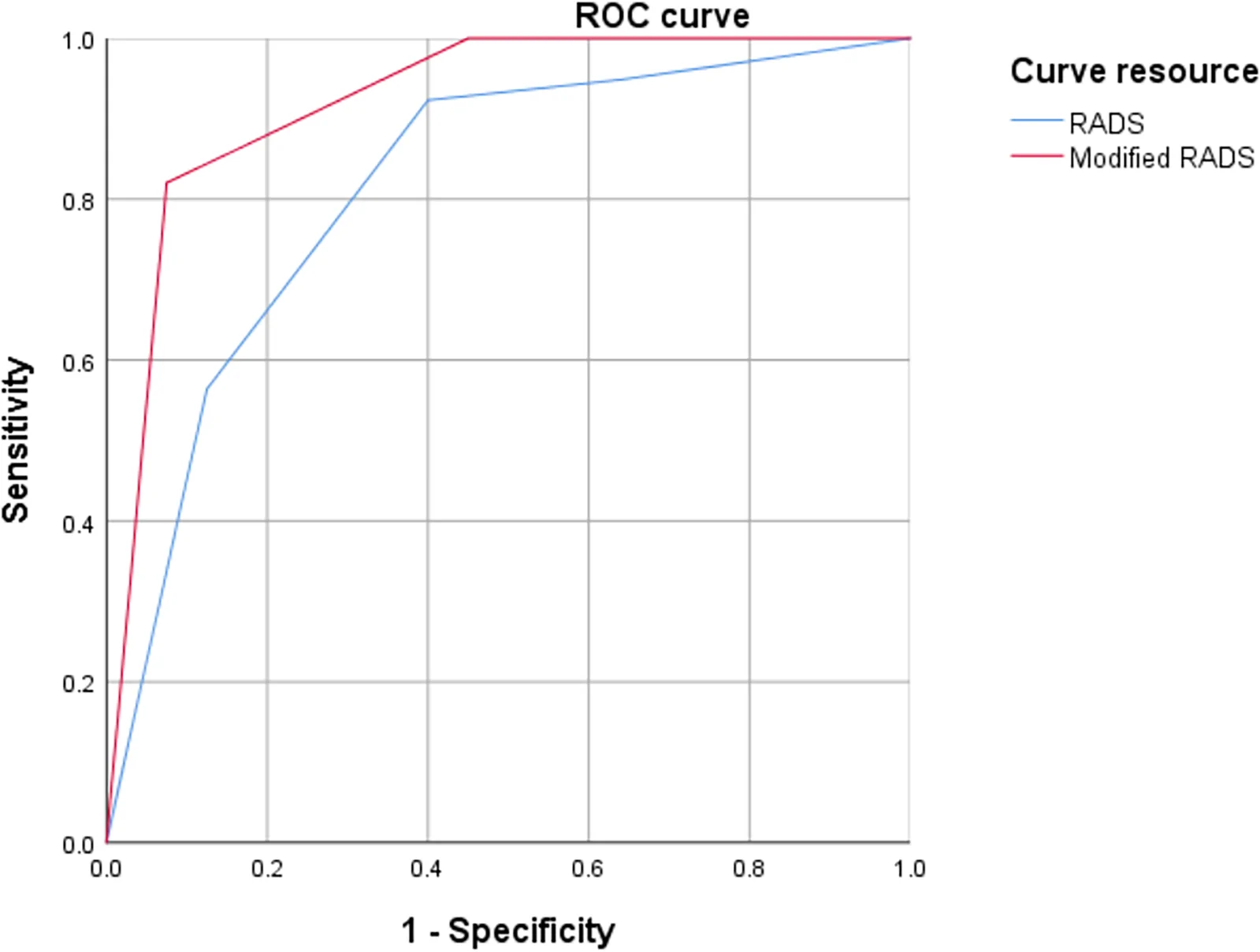
Receiver operating characteristic (ROC) curves for detection of malignancy of score 1 (O-RADS US) and score 2 (O-RADS US combined with the ROMA). The area under the curve for score 2 was higher than that for score 1 (p < 0.05).

## Discussion

Ovarian cancer (OC) remains a challenging clinical issue due to tumor heterogeneity, uncertainty in tissue origin, and the lack of reliable early detection strategies. Currently, no standardized screening tools are universally recommended for OC. While biomarkers, imaging modalities, and predictive models are widely employed to differentiate benign from malignant ovarian masses, consensus on optimal diagnostic criteria is lacking.

The O-RADS US system provides an objective risk stratification framework for ovarian masses by integrating vascular, morphological, and biological features. In contrast, the ROMA algorithm leverages serum CA-125, HE4 levels, and menopausal status to discriminate malignant from benign lesions but omits imaging parameters entirely. To address these limitations, we developed a novel scoring system by incorporating the ROMA algorithm into the ACR O-RADS US framework, aiming to refine ultrasound-based risk stratification.

Our findings revealed significant shifts in classification patterns following integration, a marked decrease in O-RADS US Category 4 lesions and substantial increase in Categories 2 and 3 lesions, however, minimal change in Category 5 lesions (23.3% vs. 23.9%). The malignancy rate for Categories 4 and 5 lesions increased from 44.0% (37/84) with the original system to 58.1% (36/62) with the modified system. Critically, the revised O-RADS US classification demonstrated superior diagnostic performance compared to both standalone ACR O-RADS US and ROMA.

Transvaginal ultrasound (TVUS) demonstrates strong diagnostic accuracy for ovarian masses with typical morphological features, particularly when integrated with the ACR O-RADS US scoring system. However, diagnostic performance declines in cases involving atypical characteristics (e.g., avascular solid components, large lesion size) or operator-dependent factors such as limited sonographer experience (14). A recent meta-analysis highlights the ACR O-RADS US system’s robust pooled sensitivity (97%) but moderate specificity (77%) for classifying adnexal masses (15).

Beyond imaging, biomarkers including CA125 and HE4, along with combined modalities, have been evaluated for early OC detection. Nevertheless, the efficacy of these methods lacks validation in prospective randomized controlled trials. While CA-125 is widely used in diagnosing OC, its utility as a standalone biomarker remains controversial due to low sensitivity and specificity, resulting in high false-negative or false-positive rates (16, 17). HE4 is also considered a suitable OC biomarker; nevertheless, an increase in HE4 is not universal among all OC types, and smoking plus contraceptive use contribute to HE4 level variations.

Accumulating evidence highlights the complexity of developing effective screening strategies for ovarian cancer, particularly for aggressive subtypes like high-grade serous carcinoma, which often originates as occult fallopian tube lesions (18). The integration of TVUS with multi-marker serum testing has shown promise in detecting both early- and advanced-stage OC, especially in high-risk populations.

The ROMA combines CA125 and HE4 measurements with menopausal status, addressing limitations of individual biomarker use. In ovarian cancer diagnosis, ROMA demonstrates 76.7%-92.3% sensitivity with 69.87-88.9% specificity in pre-menopausal women, and 73.9%-100.00% sensitivity with 74.2%-94.5% specificity in post-menopausal women (4, 19, 20). In the present study, ROMA’s sensitivity and specificity were 80.6% and 76.8% in pre-menopausal patients, and 88.0% and 78.5% in post-menopausal patients, respectively. Although the sensitivity resembles previous findings, a substantial specificity difference exists. Other studies confirm ROMA’s variable sensitivity/specificity and similar diagnostic performance between pre- and post-menopausal groups. This variability stems from geographic differences, study population sizes, disease spectra and target disease prevalence, plus analytical systems and measurement methods for HE4/CA125 across studies. However, Kaijser et al.’s meta-analysis demonstrated ROMA distinguishes ovarian carcinoma with 76%-86% sensitivity and 74%-95% specificity, regardless of marker measurement method (21). In addition, ROMA’s diagnostic utility has primarily been validated in high-risk, specialty referral cohorts—not in general or low-risk primary care populations (7).

To address this limitation, prior efforts have focused on augmenting ROMA with additional biomarkers (22, 23). In contrast, our study introduces an innovative hybrid framework that integrates ultrasound-derived morphological features (guided by O-RADS US criteria) with ROMA’s biomarker profile. This modified O-RADS classification system aims to enhance the discrimination of benign versus malignant ovarian tumors by synergizing imaging and serological data.

When integrating ROMA values into the ACR O-RADS US system, 6, 24, 40, and 13 lesions in categories 2, 3, 4, and 5 respectively underwent classification adjustments. Per the modified O-RADS US criteria, all category 3 masses downgraded to category 2 were benign. Among category 4 and 5 masses downgraded to categories 3 and 4 respectively, 1 and 6 lesions were malignant. Conversely, category 2, 3, and 4 masses upgraded to categories 3, 4, and 5 showed malignancy in 0, 0, and 7 lesions respectively. These findings further revealed that, compared with Scoring system 1, Scoring system 2 exhibited a decrease in sensitivity for diagnosing ovarian malignant tumors from 92.3% to 82.1%, whereas specificity increased from 60.0% to 92.5%. This shift may be attributed to factors influencing the expression levels of CA125 and HE4, which is consistent with the lower sensitivity and higher specificity of the ROMA index reported in both this study and previous literature (24). Notably, the modified O-RADS US system maintains a favorable balance between sensitivity and specificity, comparable to that of the original system. We state that this approach has the potential to enhance specificity and decrease the classification of lesions as intermediate risk, pending prospective clinical validation.

This study has several limitations, the most significant being methodological. The ROMA thresholds were derived from ROC analysis within the current dataset and subsequently applied to the same patient cohort to construct the modified O-RADS US classification. Consequently, the diagnostic performance of this new system was evaluated within the same sample. This approach may lead to an overly optimistic estimate of diagnostic accuracy and increase the risk of overfitting. In addition, selection bias remains a critical limitation, as classic benign lesions (e.g., simple cysts) were excluded. Prospective observational studies across diverse patient cohorts are required to validate this system’s utility in broader community settings. Finally, key limitations encompass the retrospective single-center design, limited sample size of malignant cases, absence of independent validation and interobserver assessment, potential clustering of bilateral lesions, reliance on data-driven ROMA thresholds, and the possibility of clinically significant malignant downgrading. To address these limitations, future studies should perform both internal and external validation to improve the generalizability of the findings.

## Data Availability

The original contributions presented in the study are included in the article/supplementary material. Further inquiries can be directed to the corresponding author.
